# Alterations in white matter integrity and network topological properties are associated with a decrease in global motion perception in older adults

**DOI:** 10.3389/fnagi.2023.1045263

**Published:** 2023-03-09

**Authors:** Shizhen Yan, Yuping Zhang, Xiaojuan Yin, Juntao Chen, Ziliang Zhu, Hua Jin, Han Li, Jianzhong Yin, Yunpeng Jiang

**Affiliations:** ^1^Faculty of Psychology, Tianjin Normal University, Tianjin, China; ^2^Medicine School of Rehabilitation, Henan University of Chinese Medicine, Zhengzhou, China; ^3^State Key Laboratory for Cognitive Neuroscience and Learning, Beijing Normal University, Beijing, China; ^4^Key Research Base of Humanities and Social Sciences of the Ministry of Education, Academy of Psychology and Behavior, Tianjin Normal University, Tianjin, China; ^5^The First Central Clinical College of Tianjin Medical University, Tianjin, China; ^6^Department of Radiology, People’s Hospital of Haikou, Haikou, China

**Keywords:** global motion perception, aging, white matter, structural network, TBSS

## Abstract

Previous studies have mainly explored the effects of structural and functional aging of cortical regions on global motion sensitivity in older adults, but none have explored the structural white matter (WM) substrates underlying the age-related decrease in global motion perception (GMP). In this study, random dot kinematogram and diffusion tensor imaging were used to investigate the effects of age-related reductions in WM fiber integrity and connectivity across various regions on GMP. We recruited 106 younger adults and 94 older adults and utilized both tract-based spatial statistics analysis and graph theoretical analysis to comprehensively investigate group differences in WM microstructural and network connections between older and younger adults at the microscopic and macroscopic levels. Moreover, partial correlation analysis was used to explore the relationship between alterations in WM and the age-related decrease in GMP. The results showed that decreased GMP in older adults was related to decreased fractional anisotropy (FA) of the inferior frontal-occipital fasciculus, inferior longitudinal fasciculus, anterior thalamic radiation, superior longitudinal fasciculus, and cingulum cingulate gyrus. Decreased global efficiency of the WM structural network and increased characteristic path length were closely associated with decreased global motion sensitivity. These results suggest that the reduced GMP in older adults may stem from reduced WM integrity in specific regions of WM fiber tracts as well as decreased efficiency of information integration and communication between distant cortical regions, supporting the “disconnection hypothesis” of cognitive aging.

## 1. Introduction

Global motion perception (GMP) is a fundamental visual process that refers to the ability to combine local motion signals within a visual scene into a global percept to obtain information about motion speed and direction (Narasimhan and Giaschi, 2012). For example, in a football scene, the trajectory of each player (a local moving element) constantly changes, but the audience can obtain a global precept of the entire scene by integrating the trajectory of all players (e.g., the players on the field advancing toward a team’s goal). This perceptual process plays an important role in navigation, judgment of motion speed, and avoidance of moving obstacles (Hoffman et al., 2015). One method extensively used to assess GMP is the random dot kinematogram (RDK), which consists of dots moving in various directions: signal dots move in a specific direction, while noise dots move in random directions (Pilz et al., 2017; Ward et al., 2018; Benassi et al., 2021; Joshi et al., 2021). The task is to identify the global direction of the moving dots. GMP is evaluated by measuring the individual motion coherence threshold (MCT): the minimum proportion of signal dots required to correctly identify the direction of global motion. A higher MCT indicates poorer performance and worse global motion sensitivity. Several studies have employed the RDK paradigm to study the effect of age on GMP. These studies found that older adults have reduced global motion sensitivity and significantly higher MCT than younger adults (Snowden and Kavanagh, 2006; Roudaia et al., 2010; Bower and Andersen, 2012). Several studies have shown that aging of the GMP is associated with a decreased ability to perceive hazards while driving (Wilkins et al., 2013; Lacherez et al., 2014). In addition, Yamasaki et al. (2016) reported that GMP aging may also be a predictor of cognitive decline in older adults. Thus, age-related declines in GMP are not only detrimental to the quality of life of older people, but also pose a serious threat to their safety as well.

Many studies have attempted to determine the neural mechanism underlying age-related decreases in GMP in older adults. Nevertheless, these studies have mainly focused on the effects of age-related structural and functional alterations in specific cerebral regions (Biehl et al., 2017; Ward et al., 2018; Jin et al., 2020, 2021). To date, little is known about the impact of white matter (WM) degeneration with age on GMP. WM fiber tracts are anatomical substrate underlying information transmission across various cerebral regions and are responsible for enabling information transfer between neurons and coordinating the fundamental functions of brain regions. According to the “disconnected brain” hypothesis of cognitive aging, decreases in structural and functional connectivity contribute to cognitive decline (Damoiseaux, 2017; Fjell et al., 2017; Coelho et al., 2021). More rapid higher-order cognitive functions require efficient communication across brain regions, but age-related alterations in the microstructural architecture of WM fiber tracts disrupt this communication, reducing cognitive function.

Diffusion tensor imaging (DTI) has been widely used to track alterations in WM underlying cognitive aging at a microstructural level. This method provides WM parameters that quantify and characterize the directionality and magnitude of water molecules in brain tissues and address WM integrity (Basser et al., 1994). Four parameters are commonly used to assess WM integrity: fractional anisotropy (FA), mean diffusivity (MD), axial diffusivity (AD), and radial diffusivity (RD). FA reflects the anisotropy of water molecule diffusion; MD represents the mean diffusivity of water molecules; AD reflects the diffusivity of water molecules parallel to the axon fibers; and RD indicates the diffusivity of water molecules perpendicular to the axon fibers. Higher FA values and lower MD, AD, and RD values indicate better microstructural integrity of brain tissues (Bennett and Madden, 2014). Numerous DTI studies have found a link between decreased WM integrity and cognitive impairment with age (Bennett and Madden, 2014; de Lange et al., 2016; Merenstein et al., 2021). These studies primarily concentrated on executive function, information processing speed, memory, and general cognitive ability; their findings supported the “disconnected brain” hypothesis (Sullivan and Pfefferbaum, 2007; Davis et al., 2009; Borghesani et al., 2013; Bennett and Madden, 2014; de Lange et al., 2016; Coelho et al., 2021). For example, the longitudinal study by Coelho et al. (2021) showed that FA in the corpus callosum (CC) and superior longitudinal fasciculus (SLF) significantly decreased in older adults, and this reduction in WM integrity was significantly associated with decreases in memory, executive function, and general cognitive performance.

Many researchers have investigated the neural underpinnings of GMP in WM (Csete et al., 2014; Braddick et al., 2017; Pamir et al., 2021). Using diffusion magnetic resonance imaging (dMRI), Csete et al. (2014) investigated the WM microstructure during motion detection; they observed that the local FA in specific WM regions (e.g., the left optic radiation) of adults was significantly correlated with their motion detection threshold. Advanced probabilistic tractography revealed that the SLF may be the tract that is closely associated with the GMP. Subsequently, Braddick et al. (2017) found that global motion sensitivity in children was positively correlated with FA in the right SLF and negatively correlated with FA in the left SLF. Furthermore, Pamir et al. (2021) recruited patients with impairment in the visual cortex and observed that the higher the RD in the tracts that connect the right V1 and V5 was, the worse the GMP. These studies demonstrate that GMP has neural correlates, providing indirect evidence for the hypothesis that age-related decreases in GMP may be associated with WM degradation.

In addition, researchers have not only explored changes in WM integrity but have also applied graph theoretical analysis to construct a WM structural network at a macroscopic level to quantitatively assess individual information transmission efficiency, network integration, and functional differentiation by using DTI. Graph theoretical analysis offers a global perspective that overcomes the limitations of previous research, in which each brain region was viewed as a discrete anatomical neural structure. This method provides new insights into the neural activity patterns of the brain and the connectivity mechanisms of various cognitive functions. Moreover, the quantitative topological measures of the WM structural network are sensitive to individual aging. Gong et al. (2009) applied graph theoretical analysis and that the local efficiency and overall cortical connectivity of the WM network decreased with increasing age after maturity. Moreover, older adults’ cognitive function is impacted by this reduction in information integration. With 342 healthy older adults, Wen et al. (2011) explored the relationship between the WM network and multidimensional cognition and found that discrete neuroanatomical networks were highly associated with cognitive performance in specific domains, such as processing speed and visuospatial and executive function. Thus, exploration of GMP-related WM substrates can enhance understanding of neurological changes associated with age-related decreases in GMP from the perspective of information integration and transmission efficiency.

The aim of the present study was to investigate the effects of age-related reduction in WM integrity and connectivity on GMP at WM microscopic and macroscopic levels using RDK and DTI. Previous studies have revealed that GMP mainly depends on regions of the dorsal visual stream and the parietal lobe (Biehl et al., 2017; Chaplin et al., 2017; Sousa et al., 2018; Ward et al., 2018). Our hypotheses were as follows: (1) Changes in the integrity of WM fiber tracts connecting the visual cortex to other cortical regions would be significantly correlated with changes in GMP in older adults. For example, the SLF provides bidirectional connections among the parietal, frontal, occipital, and temporal lobes and may thus play an important role in transmitting motion information from the occipital lobe to the parietal and prefrontal areas (Kamali et al., 2014); we predicted that its changes in the integrity would be correlated with changes in GMP. (2) Older adults would exhibit a reduced ability to integrate information in the WM network, as indicated by significant changes in topological properties (e.g., a significant increase in characteristic path length) that affect GMP.

## 2. Methods

### 2.1. Participants

We recruited 118 older and 113 younger adults for this study through an advertisement. The older adults were locals aged 60 years or older from Tianjin, China, and the younger adults were healthy university students. Before the formal experiment, all participants underwent screening for visual function and MRI contraindications. Vision screening was designed to assess the participants’ eye health, i.e., whether there were physiological or pathological abnormalities of the visual system such as myopia, hyperopia, amblyopia, glaucoma, cataracts and age-related macular degeneration. The younger adults completed this assessment by self-reported questionnaires, while the older adults completed it with the help of two clinicians. In addition, the mental health of older adults was assessed using the Mini-Mental State Examination (MMSE), and their brain aging and pathological abnormalities were examined by two imaging clinicians.

Twenty-one older participants were excluded from the study because of the following structural abnormalities: brain tumors, atrophy, leukoaraiosis, infarcts, and cystic lesions. A total of 3 older and 7 younger participants were additionally excluded due to head movement artifacts (ring) in structural images or geometric distortions in DTI. Finally, 106 younger adults aged 18–27 years (23.04 ± 5.17 years old, 66 female) and 94 older adults aged 60–84 years (65.74 ± 4.50 years old, 55 female) were included in this study. The final participants met the following criteria: (1) normal or corrected-to-normal vision; (2) no significant history of neurological or psychiatric disease, serious physical illness, or substance abuse; (3) no contraindications to MRI or structural brain abnormalities; and (4) older adults scored more than 24 points on the MMSE (scores range: 26 ~ 30; *M* ± *SD*: 28.80 ± 1.18) (Porter et al., 2017). All participants provided written informed consent and received payment for their participation. The study was approved by the Ethics Committees of Tianjin First Central Hospital and Tianjin Normal University.

### 2.2. RDK

The stimuli were created in MATLAB 2015a (MathWorks Inc. Natick, MA, United States), generated using a 17-inch HP Zbook17 G3 workstation, and displayed at a resolution of 1920 × 1,080 pixels (refresh rate of 60 Hz) and a mean luminance of 180 cd/m^2^. A horizontally oriented RDK paradigm was used to assess individual GMP. The stimulus was presented in a circular aperture with a diameter of 11° at the center of the black screen, and it contained 1,00 white dots. The dot diameter was 2.16 arcmin, and the dot density was 0.88 dots/cm^2^. All dots had a limited lifetime of 500 ms (equivalent to 10 frames). The position of each dot was randomly allocated at the beginning of each trial. Some of these dots (signal dots) moved horizontally to the left or right in a coherent manner. Once the dot moved out of the stimulus region, it was placed at a random position within the aperture, and set to move in the same direction as before. The other parts of dots (noise dots) are plotted in new locations, randomly selected within the display area, on each frame of the sequence. The global motion direction of each trial was randomly assigned, but the numbers of leftward and rightward trials were equal overall. The speed of the dots was set at 1°/s, 1.4°/s, or 1.8°/s to prevent potential anticipation or adaptation effects (Berry et al., 1999; Anton-Erxleben et al., 2013).

We employed a three-down/one-up (79.37% correct) adaptation staircase procedure to control the coherence level of moving dots. In other words, if three consecutive accurate responses were given, the coherence level was reduced by one step; if one incorrect response was given, it was increased by one step. For each session, there were eight reversals in coherence. The starting coherence level of the dots was 100%; the decrements had a step size of 10% (for the first two reversals) and 5% (for the third to eighth reversals). We calculated the average coherence level for the third to eighth reversals and regarded it as the global MCT for each participant. The MCT is the ratio of the minimum number of signal dots to the total number of dots required for an individual to identify the global motion direction of an RDK.

**Figure 1:**
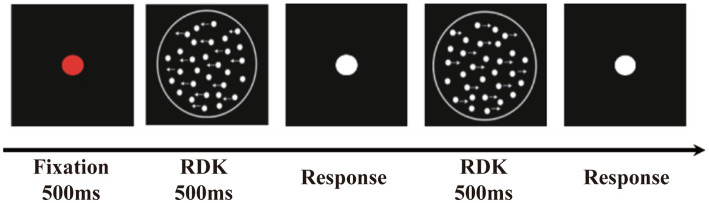
The random dot kinematogram paradigm (RDK). The stimuli contained some white luminance dots moved horizontally to the left or right coherently (signal dots) and other dots moved randomly (noise dots).

Participants sat in front of the center of the screen with a viewing distance of 60 cm. At the beginning of the sequence, a red fixation point was presented, followed by an RDK. Then, the participants were instructed to complete a two-alternative forced-choice task indicating whether the global direction of the RDK was to the left or to the right as quickly and accurately as possible. After participants responded, a white dot appeared, indicating the beginning of the next trial (Figure 1). The test consisted of six blocks, each block containing 60 trials, and was conducted in two sessions. Before the formal test, participants were given 20 practice trials to familiarize themselves with the procedure. The participants were able to control the rest time between blocks. The entire test took approximately 30 min to complete.

### 2.3. MRI data acquisition

Both older and younger adults were scanned using a 3.0 T Prisma (Siemens Healthcare, Germany) MRI scanner with a standard 64-channel head coil at the Brain Imaging Research Centers of Tianjin First Central Hospital and Tianjin Normal University, respectively. During the scan, the participants lay flat in the scanner with their heads immobilized by cushions to reduce head movement and wore earplugs to reduce noise and enhance comfort. For each participant, whole-brain anatomical data were collected using a T1-weighted 3-D MPRAGE sequence with the following parameters: echo Time (TE) = 2.98 ms, field of view (FOV) = 256 × 256 mm2, acquisition matrix size = 256 × 256, and voxel size = 3.5 mm, 176 layers, slice thickness = 1 mm, repetition time (TR) = 2,300 ms (older adults) or 2,530 ms (younger adults), and duration of approximately 8 min. DTI was performed using an echo planar imaging (EPI) sequence with the following parameters: TR = 8,500 ms, TE = 63 ms, FOV = 224 mm × 224 mm, acquisition matrix size = 112 × 112, 75 layers, slice thickness = 2 mm, b value = 1,000 s/mm^2^, 64 diffusion gradient coding directions, and duration of 10 min and 56 s. Older adults underwent two routine scans to exclude individuals with structural brain alterations: tse2d1_15: TR = 3,500 ms, TE = 89 ms, TA = 68 s, FA = 150°, slice thickness = 5 mm, FOV = 195 mm × 240 mm, matrix = 250 × 384, slices = 22. *fl2d1: TR = 250 ms, TE = 2.43 ms, TA = 70 s, FA = 85°, slice thickness = 5 mm, FOV = 195 mm × 240 mm, matrix = 250 × 384, 22 slices.

### 2.4. Data analysis

#### 2.4.1. DTI data preprocessing and tract-based spatial statistics analysis

DTI data preprocessing was performed using PANDA (Pipeline for Analyzing Brain Diffusion) software,^1^ which is an automated toolbox for dMRI analysis. In brief, the preprocessing included the following steps: (1) conversion of DICON files to NIfTI images; (2) brain tissue extraction; and (3) correction for head motion artifacts and eddy current distortions. To avoid image distortion, each image was coregistered to the b0 image. Additionally, (4) gradient orientation correction was performed based on the deformation field to estimate the tensor and fiber direction more accurately, and (5) four dMRI measures (FA, MD, AD, and RD) were calculated by fitting a diffusion tensor model.

Tract-based spatial statistics (TBSS) in FSL^2^ software was performed to enable voxelwise comparison between the groups. In brief, the analysis steps were as follows: (1) individual FA images were aligned to the mean FA standard template (FMRIB58_FA) in Montreal Neurological Institute (MNI) space using the nonlinear registration algorithms of FNIRT; (2) the mean of all aligned FA images was calculated and skeletonized to generate a WM FA skeleton, with analysis limited to major WM tracts using a threshold of FA > 0.2; and (3) individual FA values (obtained by finding the maximum value perpendicular to the local skeletal structure from the nearest skeletal center) were projected onto the mean FA skeleton. These steps were repeated to calculate individual MD, AD, and RD and project these maps onto the mean FA skeleton.

#### 2.4.2. WM network construction

After preprocessing, a WM structural network, consisting of a collection of nodes connected by edges, was constructed using PANDA. In the present study, the automated anatomical labeling 90 atlas (AAL 90) was used to define the nodes of the WM network, which included a total of 90 cortical and subcortical regions (45 for each hemisphere). To transform the AAL template in MNI space to the DTI space, where the subject data were located, we first coregistered the individual T1 structural images to b0 images with transformations. Then, a nonlinear transformation to register the aligned T1 images in MNI space to the AAL template was applied. Finally, the AAL template in MNI space was transformed into the individual DTI space using the inverse transform to locate the 90 nodes for each participant.

The edges represent the WM connectivity or features of the brain between two nodes. We used the Fiber Assignment by continuous tracking algorithm (FACT) for deterministic tractography to define the connectivity between nodes. Fiber tracking was performed using each voxel with FA greater than 0.2 as a seed point, and tracking was stopped when the turning angle exceeded 35°. Each pair of nodes was considered structurally connected if there was at least one streamline whose end points were located in the pair. The mean FA of the streamline linking the two nodes was defined as the edge and used to construct an FA-weighted matrix. Finally, the FA-weighted structural network was obtained for each participant from their DTI data, which was represented as a 90 × 90 symmetric matrix.

#### 2.4.3. Graph theoretical analysis

To characterize the topological organization of WM structural connections, topological properties were calculated by the GRETNA toolkit.^3^ The following four global topological properties were used in this study: the global clustering coefficient (Cp), characteristic path length (Lp), global efficiency (Eg), and local efficiency (Eloc). Cp is mainly used to measure the extent of local clusters or cliquishness of the network. Lp indicates the length of the shortest path of information from one node to another in the network and reflects the extent of the overall routing efficiency of a network. E.g., measures the global information propagation of the network. Eloc represents the local efficiency of the network. We focused on these global network properties to examine the efficiency of global integration and segregation of information flow. We also assessed small-world properties (λ, σ, and γ). Briefly, normalized path length (λ) is a measure that reflects the global integration of the brain, while the normalized clustering coefficient (γ) represents global segregation. Detailed calculations and interpretations of the topological properties are seen in Table 1 and Rubinov and Sporns (2010). We calculated these two properties at each sparsity threshold (0.05 ~ 0.5, step 0.05) and computed the respective area under the curve (AUC) over the range of sparsity thresholds. The ratio between segregation and integration is the small-worldness (σ) of a network. In a network, λ ≈ 1 and γ ≫ 1 suggest an optimal balance between functional segregation and integration.

**Table 1:** The mathematical definitions and descriptions of global and small-world parameters.

Network parameters	Definitions	Descriptions
Global parameters	Characteristic path length $L_{p}=\frac{1}{N\left(N-1\right)}\underset{i\neq j\in G}{\sum }L_{ij}$	*l_ij_* is the shortest absolute path length between nodes *i* and *j*. *N* is the total number of nodes and *G* is the set of all nodes. Paths are sequences of distinct nodes and links in the network to represent potential routes of information flow between pairs of brain regions.
	Clustering coefficient $Cp=\frac{1}{N}\underset{i=1}{\sum }\frac{E_{i}}{D_{nod}\left(i\right)\left(D_{nod}\left(i\right)-1\right)/2}$	*D_nod_*(*i*) is the degree of node *i*, *E_i_* is the number of edges in the subgraph of node *i* and *N* is the number of nodes in the network.
	Global efficiency $E_{g}\left(G\right)=\frac{1}{N\left(N-1\right)}\underset{i\neq j\in G}{\sum }\frac{1}{L_{ij}}$	$E_{g}$ is computed on disconnected networks. Paths between disconnected nodes are defined to have infinite length and correspondingly zero efficiency.
	Local efficiency $E_{loc}\left(G\right)=\frac{1}{N}\underset{i\in G}{\sum }E_{g}\left(G_{i}\right)$	*G_i_* denotes the subgraph composed of the nearest neighbors of node *i.*
Small-world parameters	Normalized clustering coefficient $\gamma =C_{p}^{real}/C_{p}^{rand}$	$C_{p}^{real}$ is the clustering coefficient of the real network and $C_{p}^{rand}$ is the mean clustering coefficient of 100 matched random network.
	Normalized path length $\lambda =L_{p}^{real}/L_{p}^{rand}$	$L_{p}^{real}$ is the characteristic path length of the real network and $L_{p}^{rand}$ is the mean characteristic path length of 100 matched random network.
	Small-worldness σ=γ/λ	network is said to be small-world if it satisfies λ ≈ 1 and γ > > 1, or δ = γ/λ > > 1. Small-world organization reflects an optimal balance of functional integration and segregation.

#### 2.4.4. Data analysis

The mean MCT at the three dot speeds for each participant was calculated as the individual GMP. Independent-sample t tests were used to analyze group differences in age, body mass index (BMI), and the MCT. The chi-square test was used to analyze sex differences between the two groups.

To investigate age-related alterations in WM microstructure, a permutation-based nonparametric inference was performed in Randomise^4^ to compare voxelwise differences in the FA skeleton between the younger and older groups. The number of random permutations was set to 5,000, and the threshold-free cluster enhancement (TFCE) method was used to correct for multiple comparisons. Subsequently, to explore the relationship between age-related decreases in GMP and changes in WM integrity, we applied masks to WM regions that differed significantly between groups, and partial correlation analyses between the MCT and FA in these masks were performed separately for the older and younger groups. As sex and BMI may affect individual WM integrity and the present study was not concerned with the effects of these factors, these variables were controlled as covariates (Daoust et al., 2021). The significance threshold of the partial correlation coefficient was set at *p* < 0.05, and the same TFCE method was used to correct for multiple comparisons.

We segmented the correlated regions according to the “JHU White-Matter Tractography Atlas” to visualize the location of WM fiber tracts associated with age-related decreases in GMP. We focused on only the tracts that contained at least one cluster with a voxel number greater than 100. The mean FA was extracted from the significant clusters of each tract, and a partial correlation analysis was performed between the mean FA and MCT to determine the strength of their correlation. Finally, we also extracted the AD and RD of older and younger adults in the same significant regions of tracts to investigate whether FA alterations associated with age-related decreases in GMP were impacted by changes in RD or AD. For this analysis, we first used independent-sample t tests to compare RD and AD between the older and younger groups and then performed partial correlation analyses of the MCT with RD and AD in the older and younger groups.

To examine the changes in the topological organization of the WM network with age, we first examined the group differences in small-world properties (λ, σ, and γ) and global topological properties (Cp, Lp, Eg, and Eloc) between older and younger groups using independent-sample *t* tests. Second, partial correlations between topological properties and the MCT were determined, with sex and BMI as covariates, in the older and younger groups to explore the relationship between alterations in network topology and age-related decreases in GMP. Multiple comparisons were corrected using the Bonferroni method.

## 3. Results

### 3.1. Behavioral results

The demographic information and GMP of the participants are shown in Table 2. The results showed statistically significant differences in age and BMI between the older and younger groups but no significant difference in the sex ratio. Since BMI may affect individual white matter integrity, this variable was controlled as a covariate in subsequent analyses (Daoust et al., 2021). Independent-sample t tests showed that the MCT of the older group was significantly higher than that of the younger group, indicating a decline in GMP and a decrease in global motion sensitivity with age.

**Table 2:** Demographic information and motion coherence threshold (*M* ± *SD*).

Term	Older adults (*n* = 94)	Younger adults (*n* = 106)	*t/χ^2^*	*p*	Cohen’s *d*
Age	65.74 ± 4.50	23.04 ± 5.17	61.94	<0.001	8.78
Sex (female/male)	55/39	66/40	0.29	0.59	
BMI	24.08 ± 2.91	21.42 ± 3.28	6.06	<0.001	0.86
MCT	34.37 ± 25.06	18.66 ± 12.19	5.74	<0.001	0.81

### 3.2. TBSS results

**Figure 2:**
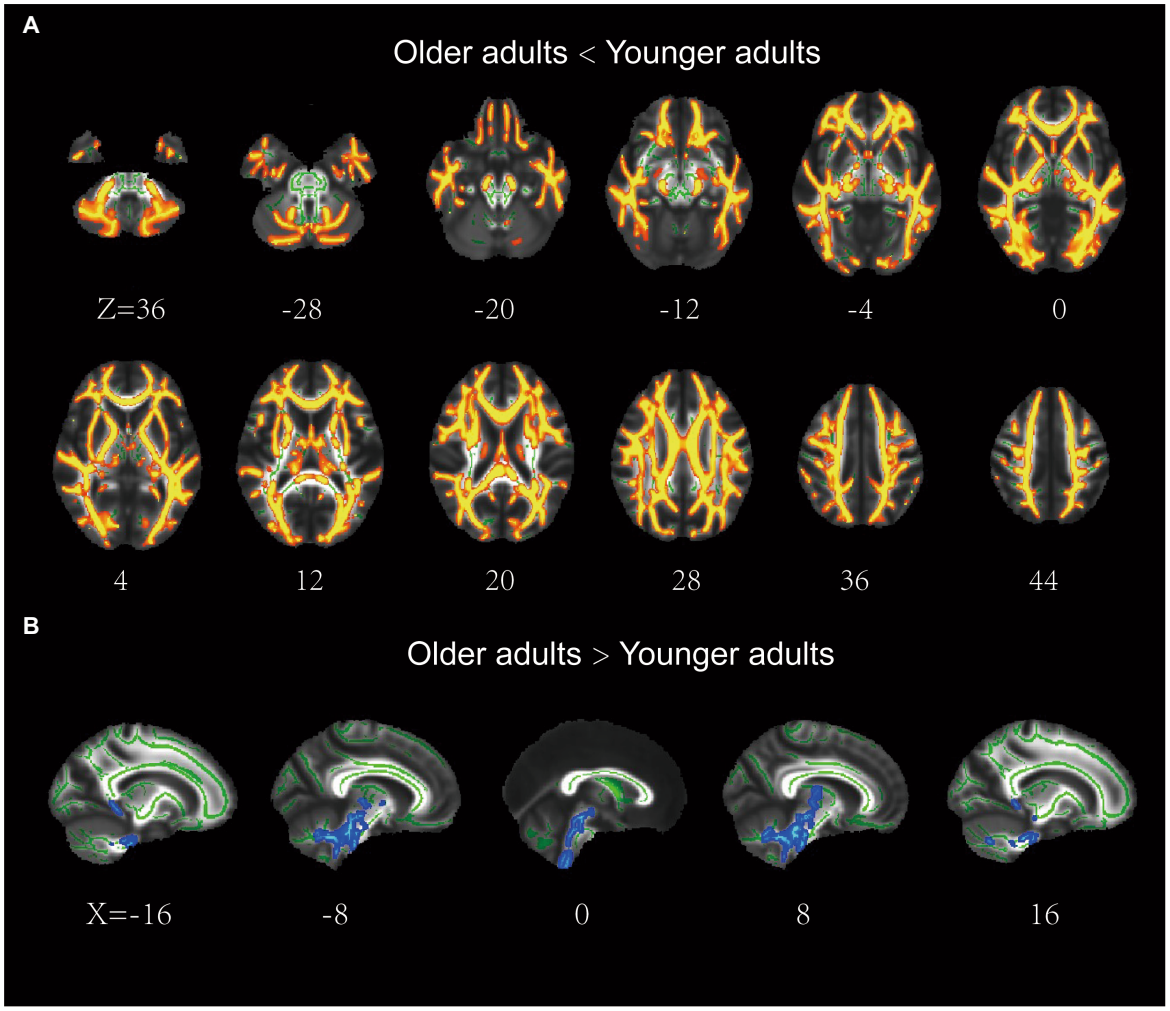
Results of the tract-based spatial statistics of fractional anisotropy between the older and younger adults. **(A)** the older adults showed lower FA than the younger adults in a large portion of the WM skeleton, including IFOF, ILF, SLF, ATR, CCG, Fmaj, and Fmin. **(B)** the older adults showed higher FA than the younger adults in a small fraction of voxels (e.g., brainstem). IFOF, inferior frontal-occipital fasciculus; ILF, inferior longitudinal fasciculus; ATR, anterior thalamic radiation; SLF, superior longitudinal fasciculus; CCG, cingulum cingulate gyrus.

The older group exhibited significantly lower FA in most regions of the WM skeleton than the younger group, suggesting that integrity in most WM regions decreases with age. In addition, a small fraction of voxels exhibited a significant increase in FA in older adults (Figure 2).

**Figure 3:**
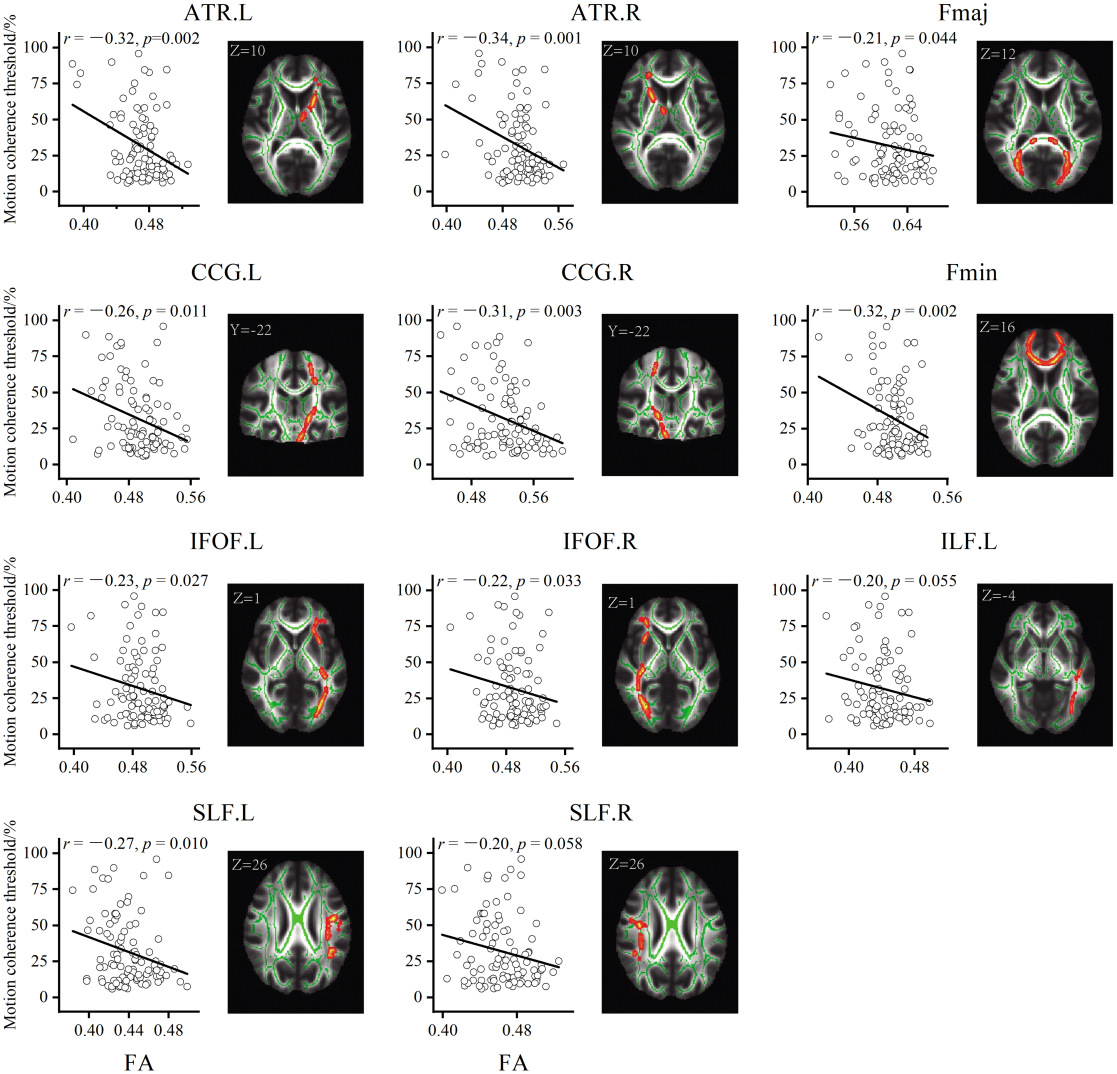
Results of the partial correlation analyses between FA and MCT in older adults. All correlations were significant at *p* < 0.05 (threshold-free cluster enhancement corrected). Decreased fractional anisotropy in the bilateral SLF, IFOF, ATR, CCG, and left ILF was associated with worse GMP.

For older adults, partial correlation analysis showed that the MCT was significantly negatively correlated with FA in four main clusters (Table 3). The effects were spread over large portions of the WM skeleton, including the bilateral CC forceps major (Fmaj) and minor (Fmin), inferior frontal-occipital fasciculus (IFOF), inferior longitudinal fasciculus (ILF), anterior thalamic radiation (ATR), SLF, and cingulum cingulate gyrus (CCG). We located the significant regions using the “JHU White-Matter Tractography Atlas,” and the voxel size of each fiber tract is shown in Table 4. We did not find any significant positive correlation between the MCT and FA in the WM regions that differed significantly between groups. We extracted the average FA in the regions of each WM tract that showed significant negative correlations with MCT and plotted the correlation between FA and the MCT to visualize the correlation strength. With sex and BMI included as covariates, the MCT was significantly negatively correlated with FA in the Fmin (*r* = −0.32, *p* = 0.002), Fmaj (*r* = −0.21, *p* = 0.044), left ATR (*r* = −0.32, *p* = 0.002), right ATR (*r* = −0.34, *p* = 0.034), left CCG (*r* = −0.26, *p* = 0.011), right CCG (*r* = −0.31, *p* = 0.003), left IFOF (*r* = −0.23, *p* = 0.027), right IFOF (*r* = −0.22, *p* = 0.033), and left SLF (*r* = −0.27, *p* = 0.010). The FA in the right SLF (*r* = −0.20, *p* = 0.058) and left ILF (*r* = −0.20, *p* = 0.055) was marginally significantly correlated with the MCT (Figure 3). In the young adults, there was no significant correlation between the MCT and FA in the WM regions that differed significantly between groups.

**Table 3:** The regions in the WM skeleton that negatively related to motion coherence threshold in older adults.

Regions	MNI coordinates of peak			Size (voxel)
	X	Y	Z	
Cluster1	4	4	24	8,694
Cluster2	−26	−70	24	1,282
Cluster3	31	−15	50	469
Cluster4	40	25	16	260

Only significant regions thresholded at *p* = 0.05, TFCE corrected.

**Table 4:** The tracts that positively corelated to motion coherence threshold.

Tracts	MNI coordinates of peak			Size (voxel)
	X	Y	Z	
Fmin	−12	46	−15	3,356
IFOF.R	35	−54	−3	1,087
IFOF.L	−28	13	−5	840
ILF.L	−41	1	−32	588
ATR.L	−10	−3	−3	498
Fmaj	30	−69	8	368
ATR.R	11	−3	−6	357
SLF.L	−52	−2	16	324
SLF.R	35	−35	24	208
CCG.L	−4	−25	−35	197
CCG.R	22	−30	42	162

**Figure 4:**
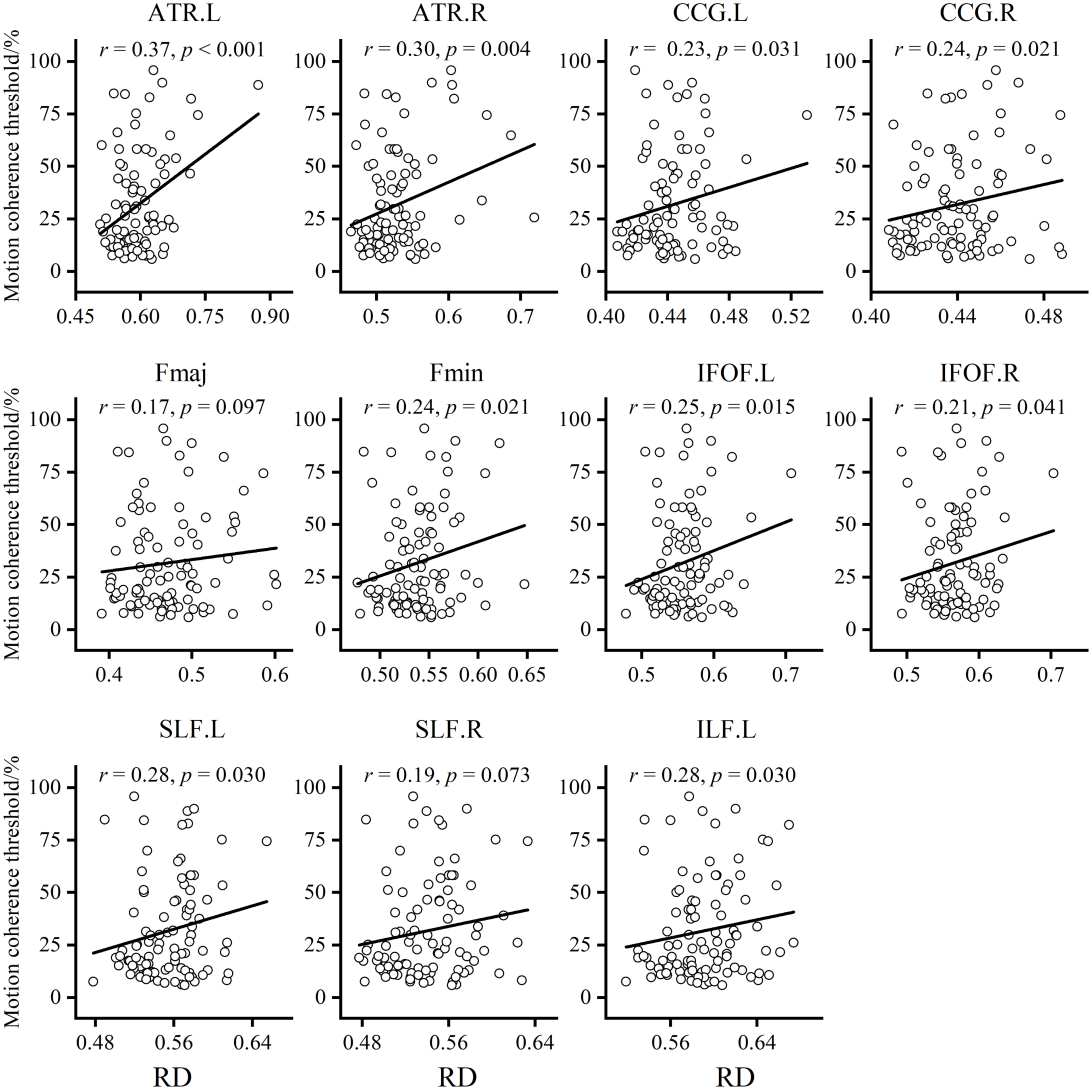
Results of the partial correlation analysis between RD and MCT in older adults. Except for Fmaj and right SLF, mean RD in most tracts showed a positive correlation with MCT of the older adults.

To explore the underlying causes of FA alterations leading to GMP decline, we additionally extracted the mean AD and RD in the relevant regions of each tract. The results showed that both the AD and RD in each tract in older adults were significantly greater than those in younger adults. The results of partial correlation analysis with sex and BMI as covariates showed that, except for the Fmaj and right SLF, the mean RD in most tracts was positively correlated with the MCT in older adults (Figure 4). However, the MCT was only positively correlated with the AD in the left ATR (*r* = 0.30, *p* = 0.003).

### 3.3. Results of the graph theoretical analysis

**Figure 5:**
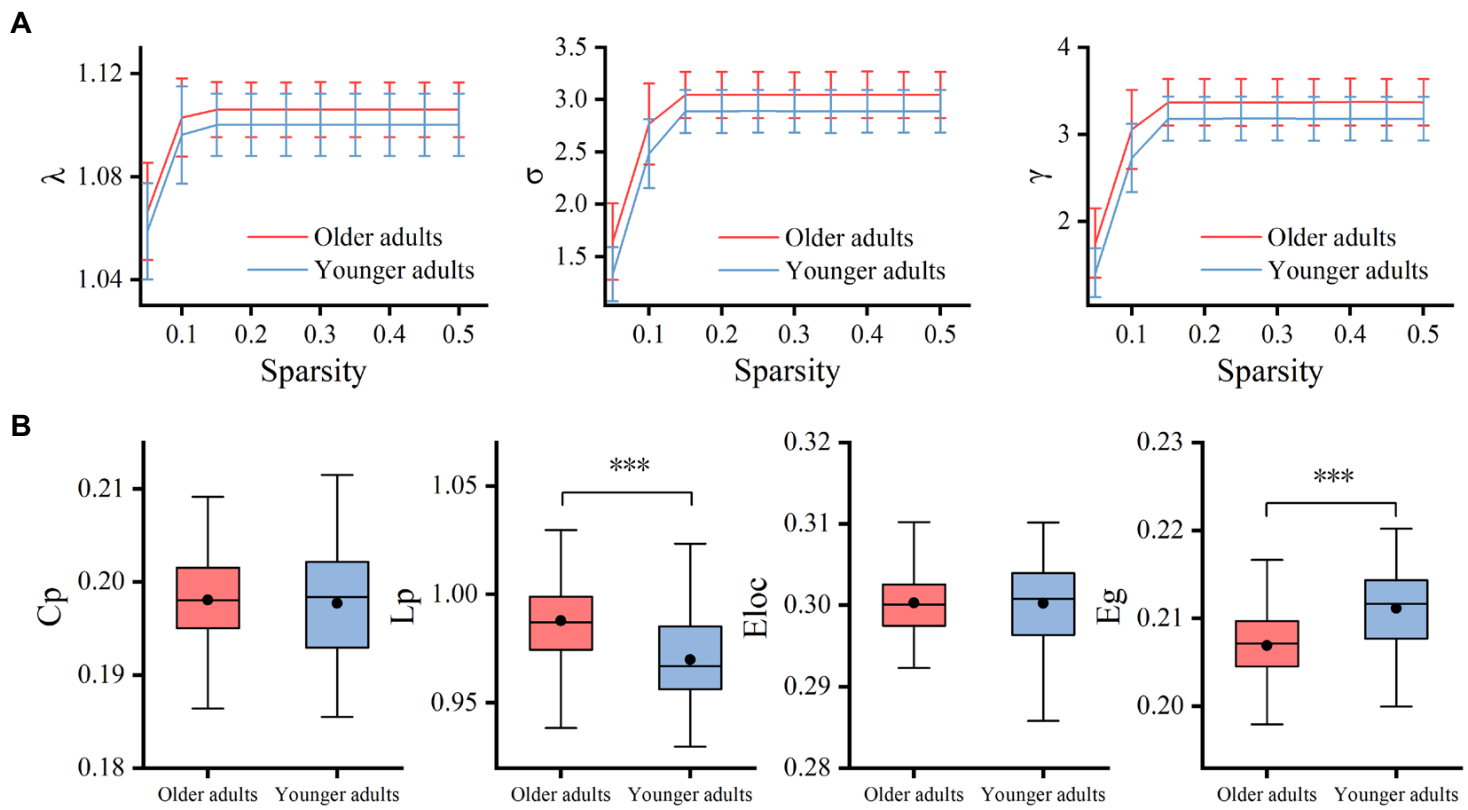
Group differences between small-world properties (λ, σ, γ) and global topological properties (Lp, Cp, Eloc, and Eg) of the WM network. 𝜆≈1 and *γ*> > 1 indicated both the older and younger adults exhibited economic “small-world” properties **(A)**. The older adults showed increased Lp and decreased Eg compared to younger adults. There was no significant difference in Cp and Eloc between the two groups **(B)**. Cp, the global clustering coefficient; Lp, characteristic path length; Eg, global efficiency; Eloc, local efficiency; λ, normalized path length; γ, normalized clustering coefficient; σ, small-worldness, ***p < 0.001.

The results showed that the WM networks of both the older and younger adults exhibited “small-world” properties (𝜆≈1, *γ*> > 1) (Figure 5A). Small-world networks have high local and global efficiency, requiring minimal connectivity costs and resulting in a balance between local processing and global integration (Watts and Strogatz, 1998). In addition, both global efficiency and the characteristic path length were found significantly decreased in older adults compared to younger adults, as shown in Table 5 and Figure 5B. Therefore, these results confirm that significant network changes occur in older adults. The results of partial correlation analyses revealed that the MCT was significantly negatively correlated with global efficiency (*r* = −0.29, *p* = 0.005) and significantly positively correlated with characteristic path length (*r* = 0.32, *p* = 0.002) in older adults, and these results survived correction for multiple comparisons. However, we did not find any significant correlations between the MCT and topological properties in younger adults.

**Table 5:** Comparisons of global topological properties between groups (×10^−1^).

	Older adults (*n* = 94)	Younger adults (*n* = 106)	*t*	*p*	Cohen’s d
Eg	2.07 ± 0.05	2.11 ± 0.05	−6.34	<0.001	−0.90
Eloc	3.00 ± 0.04	3.00 ± 0.05	0.05	0.957	0.01
Cp	1.98 ± 0.05	1.98 ± 0.05	0.41	0.680	0.06
Lp	9.88 ± 0.21	9.70 ± 0.21	6.07	<0.001	0.86

## 4. Discussion

In the present study, we attempted to explain the age-related decline in GMP according to age-related WM changes in the brain. TBSS analysis and graph theoretical analysis of dMRI data were used to assess WM integrity and construct structural networks that represented the anatomical connectivity of the cerebral cortex at the microscopic and regional levels. The behavioral findings, with the older group exhibiting significantly higher MCT values than the younger group (indicating a decrease in GMP with age), are consistent with previous literature (Biehl et al., 2017; Conlon et al., 2017; Meier et al., 2018; Mikellidou et al., 2018). According to the correlation analyses, the decreased GMP in older adults was associated with decreased WM integrity in specific tracts as well as alterations in characteristic path length and global efficiency in the WM network. The findings support the “disconnection hypothesis” of the aging brain with data on the transmission efficiency of the WM network.

### 4.1. Microstructural changes in WM associated with age-related decreases in GMP

Older adults showed decreased FA in the majority of WM compared to younger adults, indicating that WM integrity decreased with age; this finding is consistent with the literature (Benitez et al., 2018; Molloy et al., 2021; Dhiman et al., 2022). More importantly, the results revealed correlations between the decrease in global motion sensitivity and decreased WM integrity in the IFOF, ILF, SLF, ATR, CCG, Fmaj, and Fmin. The CC is the largest commissural fiber, and the main WM tract connects the two hemispheres of the brain. This tract is crucial for the transmission of sensory, motor, and cognitive information between the brain hemispheres (Aboitiz et al., 1992; Gazzaniga, 2000). The Fmaj is the callosal tract that connects the bilateral occipital lobes through the splenium. In motion processing, the Fmaj is associated with visuomotor integration between the two hemispheres (Miller, 1991; Mordkoff and Yantis, 1991; Tamura et al., 2007). In particular, the Fmaj directly connects the right and left V5 (Strong et al., 2019), a crucial area for global motion processing that is responsible for integrating dynamic local visual information into a global percept (Newsome and Pare, 1988; Britten et al., 1993; Rust et al., 2006; Chakraborty et al., 2017). The Fmin refers to the callosal tract that passes through the rostrum and genu of the CC and bends forward to connect the right and left frontal lobes. The dorsolateral prefrontal cortex of the frontal lobe participates in top-down motor control (Kim and Shadlen, 1999), while the medial prefrontal cortex may be associated with spatial working memory in self-navigation (Sherrill et al., 2015). These results suggest that decreased efficiency of information transmission between the right and left frontal and occipital lobes, especially between the right and left V5, may contribute to the reduction in GMP in older adults.

The SLF, ILF, and IFOF are association fibers. The SLF is a bundle that connects the frontal, occipital, parietal, and occipital lobes on the ipsilateral side; the ILF connects the visual areas in the temporal and occipital areas to the amygdala and hippocampus; and the IFOF is the longest association fiber in the brain, linking the frontal and occipital lobes. These tracts connect the occipital lobe to ipsilateral cortical areas and are thought to be associated with spatial information processing (Vaessen et al., 2016). For example, long-term sports training (e.g., table tennis, gymnastics) can increase the structural integrity of these three tracts (Huang et al., 2015; Qi et al., 2021), while brain injuries to these tracts affect visuospatial processing (Chechlacz et al., 2012; McGrath et al., 2013; Hattori et al., 2018). In the present study, we found that decreased WM integrity in the SLF, ILF, and IFOF leads to a decline in GMP (in terms of behavioral performance) in older adults, consistent with previous findings in region of interest (ROI) analyses that GMP is strongly related to the SLF in adults and children (Csete et al., 2014; Braddick et al., 2017). We suggest that the correlation between association fiber integrity and GMP may be due to GMP requirements. The GMP involves processing of local movement across the visual field and integrating local moving elements into a global percept; these two processes depend on the primary visual cortex, located in the occipital lobe, and the middle temporal gyrus, located in the temporal lobe, respectively (Britten et al., 1993; Rust et al., 2006). In the RDK task, participants need to make decisions about the direction of motion; these decisions mainly depend on the intraparietal sulcus, located in the parietal lobe (Kayser et al., 2010). Lack of structural connections between the occipital lobe and other cortices may cause a decrease in motion sensitivity in older adults, explaining why alterations in the SLF, ILF, and IFOF lead to impaired GMP.

Decreased FA in the ATR and CCG was also associated with decreased GMP. The CCG plays a critical role in cognitive control, conflict monitoring in response selection, and spatial attentional control (Morecraft et al., 1993, 2012; Nieuwenhuis et al., 2003). The reduced WM integrity in the CCG of older adults may thus affect spatial attentional control and decision-making processes involving motion direction in GMP. Additionally, thalamic neurons receive sensory and motion information from the external environment and transmit information to the cerebral cortex through the ATR (Perani et al., 2021). The reduced WM integrity of the ATR may have a negative influence on the transmission of visuomotor information to the cerebral cortex, leading to a reduction in GMP.

In addition, we found that most RD values of GMP-related tracts were significantly correlated with the MCT, which further indicated that the decrease in WM integrity may be caused by the increased RD of tracts in older adults. Different patterns of WM changes have been examined to elucidate aging processes in different tracts and their underlying biological profiles. Molloy et al. (2021) identified five main patterns of overlap between diffusion measures in WM areas that showed age-related negative correlations with FA. Consistent with the results reported by Molly et al., we also found decreased FA and increased RD in the Fmaj, Fmin, SLF, ILF, IFOF, and CCG, consistent with the “FA and RD only” pattern. This pattern may reflect age-related demyelination of WM tracts (Sullivan and Pfefferbaum, 2007; Molloy et al., 2021).

### 4.2. Changes in topological properties of WM networks associated with age-related decreases in GMP

In the WM structural network, global efficiency and the characteristic path length are commonly used measures of functional integration, which refers to the ability to rapidly combine specialized information from distributed cortical regions. The shorter the path length and the higher global efficiency of a network the higher the efficiency of information transmission across network nodes (Watts and Strogatz, 1998). Local efficiency and the global clustering coefficient are considered indicators of network segregation in the brain. Network segregation is the ability of densely interconnected groups of brain regions to perform specialized processing and is thought to reflect the local information transmission of the network (Latora and Marchiori, 2001; Rubinov and Sporns, 2010). Thus, our findings showed that the global efficiency of older adults was significantly lower than that of younger adults, while the characteristic path length of older adults was significantly higher; however, we found no significant difference in local efficiency or the global clustering coefficient between the two groups, indicating that the changes in the connectivity of older adults may reduce parallel information processing and the speed and efficiency of information transmission across brain regions (Achard et al., 2012). Moreover, the age-related decrease in global efficiency and the increase in characteristic path length were closely related to the reduction in global motion sensitivity in older adults, suggesting that the reduction in global motion sensitivity in older adults may be affected by the decrease in information integration and communication efficiency between distant cortical regions. Long-range connectivity (e.g., between the frontal and occipital lobes) is thought to play an important role in visuospatial attention, which is a prerequisite for global motion processing (Barceló et al., 2000; Ruff et al., 2006). Therefore, reduced efficiency of information integration and communication between distant cortical regions in older adults may lead to decreased GMP by affecting individual visuospatial attention.

This study has some limitations. First, aging is a lifelong process, but we selected participants with two discrete age ranges; thus, we were unable to explore the relationship of alterations in WM microstructure and network properties with perceptual changes caused by aging along a continuum. Second, this study mainly examined the alterations in WM integrity in 20 main tracts; we did not assess changes in structural connectivity along the dorsal visual stream (such as the connections between V1 and V5). In previous studies, patients with cortical visual impairments exhibited worse GMP, suggesting that structural disconnection along the dorsal visual stream may also cause a decrease in motion sensitivity in older adults (Pamir et al., 2021). In the future, we plan to explore the effects of structural disconnection along the dorsal visual stream on global motion sensitivity in older adults.

## 5. Conclusion

In this study, we used RDK and DTI to study the effects of age-related reductions in WM fiber integrity and connectivity on the decrease in GMP at the microscopic and macroscopic levels. We found that reduced WM integrity in specific fiber tracts, such as the Fmaj, Fmin, ILF, SLF, ATR, IFOF, and CCG, may underlie age-related decreases in GMP in older adults. Moreover, age-related decreases in GMP may also be associated with reduced information integration and communication efficiency between distant cortical areas. This study demonstrated, for the first time, that age-related reductions in WM integrity and connectivity in older adults affect the efficiency of information transfer between brain regions, leading to a decrease in global motion sensitivity. Our results thus support the “disconnection hypothesis” of cognitive aging.

## Data availability statement

The raw data supporting the conclusions of this article will be made available by the authors, without undue reservation.

## Ethics statement

The studies involving human participants were reviewed and approved by Ethics Committees of Tianjin First Central Hospital and Tianjin Normal University. The patients/participants provided their written informed consent to participate in this study.

## Author contributions

SY, YZ, and HJ gave study conceptualization and design. SY, XY, JC, ZZ, HL, JY, and YJ were involved in data collection. SY and HJ helped with data analysis and interpretation. SY, YZ, and HJ contributed to the supervision of the study procedures. SY and HJ contributed to drafting the manuscript. All authors contributed to the article and approved the submitted version.

## Funding

This work was supported by grants from the National Natural Science Foundation of China (31971021) and Tianjin Postgraduate Research Innovation Project (2019YJSB129).

## Conflict of interest

The authors declare that the research was conducted in the absence of any commercial or financial relationships that could be construed as a potential conflict of interest.

## Publisher’s note

All claims expressed in this article are solely those of the authors and do not necessarily represent those of their affiliated organizations, or those of the publisher, the editors and the reviewers. Any product that may be evaluated in this article, or claim that may be made by its manufacturer, is not guaranteed or endorsed by the publisher.

## Footnotes

^1^ http://www.nitrc.org/projects/panda

^2^ http://www.fmrib.ox.ac.uk/fsl/tbss/index.html

^3^ http://www.nitrc.org/projects/gretna/

^4^ https://fsl.fmrib.ox.ac.uk/fsl/fslwiki/Randomise
